# Survival among patients with relapsed/refractory diffuse large B cell lymphoma treated with single-agent selinexor in the SADAL study

**DOI:** 10.1186/s13045-021-01122-1

**Published:** 2021-07-16

**Authors:** Marie Maerevoet, Josee M. Zijlstra, George Follows, Rene-Olivier Casasnovas, J. S. P. Vermaat, Nagesh Kalakonda, Andre Goy, Sylvain Choquet, Eric Van Den Neste, Brian Hill, Catherine Thieblemont, Federica Cavallo, Fatima De la Cruz, John Kuruvilla, Nada Hamad, Ulrich Jaeger, Paolo Caimi, Ronit Gurion, Krzysztof Warzocha, Sameer Bakhshi, Juan-Manuel Sancho, Michael Schuster, Miklos Egyed, Fritz Offner, Theodoros P. Vassilakopoulos, Priyanka Samal, Matthew Ku, Xiwen Ma, Kelly Corona, Kamal Chamoun, Jatin Shah, Sharon Shacham, Michael G. Kauffman, Miguel Canales

**Affiliations:** 1 Service Hématologie, Institut Jules Bordet, 1000 Brussels, Belgium; 2 Amsterdam UMC, Vrije Universiteit, Cancer Center, Amsterdam, Netherlands; 3 Addenbrooke’s Hospital, Cambridge, UK; 4 Hématologie Clinique and INSERM 1231, CHU Dijon, Dijon, France; 5 LUMC, Leiden, Netherlands; 6 University of Liverpool, Liverpool, UK; 7 Hackensack University Medical Center, Hackensack, USA; 8 Hôpital Pitié Salpêtrière, Paris, France; 9 Cliniques Universitaires Saint-Luc, Brussels, Belgium; 10 Cleveland Clinic, Cleveland, USA; 11 APHP, Hemato-oncology, Saint-Louis Hospital, Paris, France; 12 Diderot University, Paris, France; 13 University of Torino, Turin, Italy; 14 Hospital Universitario Virgen del Rocio, Sevilla, Spain; 15 Princess Margaret Cancer Centre, Toronto, Canada; 16 St. Vincent’s Hospital Sydney, Darlinghurst, Australia; 17 Medical University of Vienna, Vienna, Austria; 18 UH Seidman Cancer Center, Cleveland, USA; 19 Rabin MC, Petah Tiqwa, Israel; 20 Tel Aviv University, Tel Aviv, Israel; 21 Instytut Hematologii I Transfuzjologii, Warsaw, Poland; 22 Dr. B. R. A. Institute Rotary Cancer Hospital, New Delhi, India; 23 Hospital Universitari Germans Trias I Pujol, Barcelona, Spain; 24 Stony Brook University Hospital Cancer Center, Stony Brook, USA; 25 Teaching Hospital Mór Kaposi, Kaposvár, Hungary; 26 UZ Gent, Gent, Belgium; 27 Laikon General Hospital, National and Kapodistrian University of Athens, Athens, Greece; 28 Institute of Medical Sciences and SUM Hospital, Bhubaneswar, Odisha India; 29 St.Vincent’s Hospital Melbourne, Fitzroy, Australia; 30 Karyopharm Therapeutics, Newton, USA; 31 Hospital Universitario La Paz, Madrid, Spain

**Keywords:** Selinexor, Exportin-1, SINE compounds, DLBCL

## Abstract

Patients with RR DLBCL who have received ≥ 2 lines of therapy have limited treatment options and an expected overall survival (OS) of < 6 months. The SADAL study evaluated single-agent oral selinexor in patients with RR DLBCL and demonstrated an overall response rate (ORR) of 29.1% with median duration of response (DOR) of 9.3 months. The analyses described here evaluated a number of subpopulations in order to understand how response correlates with survival outcomes in order to identify patients who could most optimally benefit from selinexor treatment. Median age was 67 years; 44.8% of patients were ≥ 70 years of age. The median OS was 9.0 months (95% CI 6.2, 13.7) at a median follow-up of 14.8 months. The median OS was not reached in patients with a CR or PR, while patients who did not respond have a median OS of 4.9 months (*p* < 0.0001). Patients < 70 years had an OS of 11.1 months compared with 7.8 months in patients ≥ 70 years. Among patients with or without prior ASCT, the median OS was 10.9 and 7.8 months, respectively. Among patients with disease refractory to the most recent DLBCL treatment regimen, the median OS was 7.0 months compared with 11.1 months for disease not refractory to the most recent treatment. In a patient population in which survival is expected to be < 6 months, treatment with single-agent oral selinexor was associated with a median survival of 9 months. Increased median OS observed in patients responding to selinexor was consistent across subgroups regardless of age, prior ASCT therapy, or refractory status. Randomized studies of selinexor in combination with a variety of other anti-DLBCL agents are planned. This trial was registered at ClinicalTrials.gov (NCT02227251) on August 28, 2014. https://clinicaltrials.gov/ct2/show/NCT02227251.

**Supplementary Information:**

The online version contains supplementary material available at 10.1186/s13045-021-01122-1.

## To the editor

Despite recent advances, nearly 50% of patients diagnosed with diffuse large B cell lymphoma (DLBCL) will succumb to their disease, with older age and comorbidities increasing risk of death and a median OS (~ 6 months) with relapsing disease after ≥ 2 prior therapies [1, 2]. XPO1 inhibition by selinexor, a first-in-class selective inhibitor of nuclear export compound results in cell cycle arrest; cells with DNA damage, including cancer cells, undergo apoptosis while sparing normal cells [3, 4]. Single-agent oral selinexor is approved for treatment of patients with DLBCL after ≥ 2 prior therapies [5]. Here, we have analyzed subgroups from the SADAL trial to understand how response correlates with survival outcomes following selinexor treatment.

Overall median OS was 9.0 months after a median follow-up of 14.8 months (95% CI: 13.2,21.7). Median OS in patients < 70 trended longer than patients > 70 (11.1 vs 7.8 months); HR 0.72 (0.46,1.13), *p* = 0.155. Patients with a best response of CR or PR on selinexor had a markedly longer median OS of 29.7 months, compared to those who did not respond (4.9 months) (*p* < 0.0001) (Table 1). Patients with lower baseline R-IPI (0–2) compared to R-IPI (3–5) had a significantly longer median OS (15.1 vs 4.6 months; HR 0.38 [0.24, 0.60], *p* < 0.0001) (Additional file 1: Figure S1). The majority (72%) of responding patients had lower R-IPI scores at baseline.

**Table 1:** Overall survival in subgroups

Patients (*n*)	All patients	Patients with CR or PR	Non-responders	HR (95% CI); *p* value*
	*N* = 134	*N* = 39	*N* = 95	
		Median (95% CI)		
All patients		NR (29.7, NR)	4.9 (4.1, 7.0)	< 0.0001
*Age*				
< 70 (*n* = 74)	11.1 (5.4, 28.0)	NR (NR, NR)	4.9 (3.1, 7.0)	0.0771 (0.0235,0.2527) < 0.0001
≥ 70 (*n* = 60)	7.8 (6.1, 13.7)	29.7 (9.1, NR)	4.6 (4.1, 12.2)	0.1912 (0.073,0.5003) 0.0002
*Region*				
North America (*n* = 20)	7.6 (4.8, 32.3)	29.7 (9.0, 29.7)	4.8 (1.6, 32.3)	0.2775 (0.0744,1.0356) 0.0430
Western Europe and Australia (*n* = 91)	10.9 (6.6, 15.5)	NR (NR, NR)	4.6 (3.0, 7.8)	0.0994 (0.0392,0.2524) < 0.0001
Central and Eastern Europe and India (*n* = 23)	6.2 (5.2, NR)	NR (6.2, NR)	5.4(3.1, NR)	0.2248 (0.028,1.8032) 0.1241
*Baseline prognosis*				
Very good (R-IPI = 0) or good (R-IPI = 1,2) (*n* = 69)	15.1 (10.9, NR)	NR (29.7, NR)	7.0 (5.2, 15.1)	0.1511 (0.0613,0.3724) < 0.0001
Poor (R-IPI = 3, 4, 5) (*n* = 58)	4.6 (3.0, 9.0)	NR (9.0, NR)	4.1 (2.5, 5.1)	0.2176 (0.0667,0.7099) 0.0056
*Number of prior systemic treatment regimens*				
2 (*n* = 79)	9.1 (5.4, 15.1)	NR (NR, NR)	4.6 (3.0, 11.1)	0.131 (0.0509,0.3372) < 0.0001
> 2 (*n* = 55)	8.2 (5.1, 29.7)	29.7 (29.7, NR)	4.9 (3.9, 7.6)	0.1389 (0.0483,0.3994) < 0.0001

*CI* confidence interval, *CR* complete response, *NR* not reached, *OS* overall survival, *PD* progressive disease, *PR* partial response, *SD* stable disease

^*^HR and *p* value is comparing responder versus non-responder

**Fig. 1:**
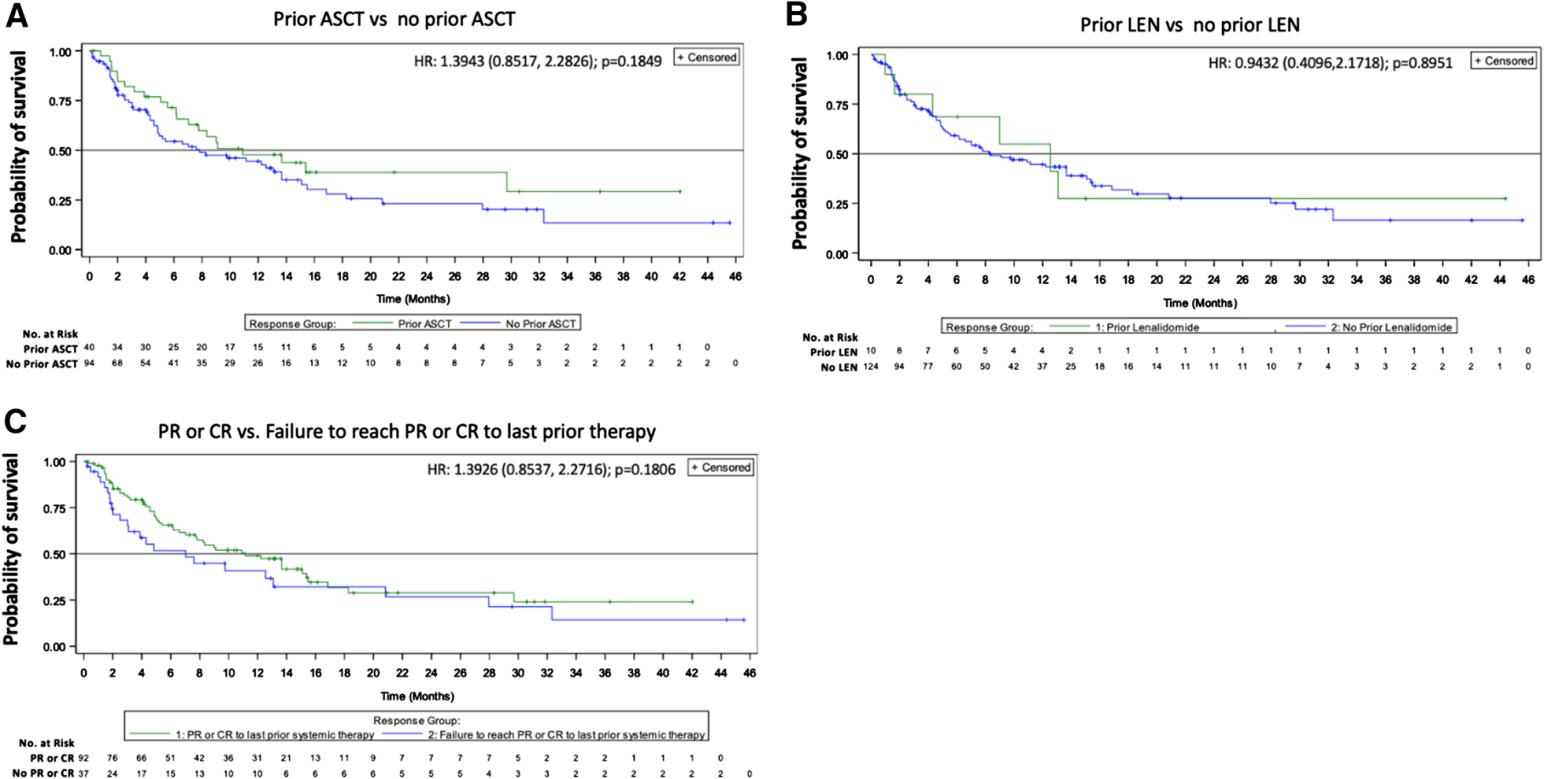
Overall survival by prior regimens and response. Kaplan–Meier curves according to **A** prior ASCT status; **B** prior use of lenalidomide; and **C** response to prior therapy: PR or CR and failure to reach PR or CR

Patients who received selinexor after ASCT compared to those who did not had a median OS of 10.9 and 7.8 months, respectively; HR 1.39 (0.85,2.28), *p* = 0.185. Regarding response to most recent systemic therapy, patients who had a CR or PR trended toward longer OS (HR 0.71 [0.44, 1.17], *p* = 0.18; medians 11.1 and 7 months) than those who did not respond (Fig. 1; Additional file 1: Table S2). A significantly shorter median OS was observed in patients with relapse < 1 year from diagnosis (5.2 vs 13.1 months). Median OS was 6.6 and 10.9 months in patients that had relapsed/refractory disease < 6 or ≥ 6 months from last use of rituximab, respectively (HR = 1.30, *p* = 0.30) (Additional file 1: Figure S2). Four patients who had not achieved CR on their most recent prior therapy achieved CR on selinexor and two patients who did not have a CR or PR to any prior therapy had a CR (*n* = 2) or PR (*n* = 2) with selinexor. Median PFS for all patients was 2.6 months (summarized for subgroups-Additional file 1: Table S1).

The median OS associated with selinexor is consistent across the majority of the analyzed subgroups and also with the novel mechanism of action and the lack of apparent cross-resistance with this and other mechanisms. Results corroborate a retrospective study on patients with RR DLBCL after ASCT, which showed that the median OS was 6.6 months with cytotoxic chemotherapy compared to 11.3 months with novel agents [6]. While combination tafasitamab and lenalidomide showed an ~ 60% ORR for relapsed/refractory DLBCL and median OS was NR after a median follow-up of 19.6 months [7], it is difficult to compare to SADAL since 50% of patients had only one prior line of therapy compared to 3% on SADAL. In addition, only 18% of patients had primary refractory disease (relapse < 6 months of frontline therapy) compared to 47% of patients with available data on SADAL. Furthermore, 44% were refractory (i.e., relapsed < 6 months) to most recent therapy compared to 66.4% of SADAL patients. Further demonstration of the efficacy and safety consistent with the novel mechanism of action and lack of cross-resistance is observed with combination treatment of selinexor with backbone chemotherapy: 100% ORR with 90% CR using R-CHOP + selinexor as frontline treatment for DLBCL and follicular lymphoma [8] and 78% ORR using R-ICE + selinexor for relapsed/refractory DLBCL [9]. These combination results are consistent with a significant anti-DLBCL contribution of selinexor to standard chemotherapy and are being further evaluated.

Single-agent oral selinexor treatment was associated with a longer OS than expected based on contemporary case series [10–12] despite patient treatment, response history, age, and comorbidities. Given the beneficial impact of selinexor as a single agent and the poor prognosis of many patients, randomized studies of selinexor in combination with a variety of other anti-DLBCL agents are planned. Taken together, selinexor represents a safe, orally available option for patients whose disease has relapsed or is refractory to ≥ 2 prior therapies, including patients > 70 years old or those with significant comorbidities.

## Supplementary Information

**Additional file 1.** Supplemental Material.

## Data Availability

Karyopharm Therapeutics agrees to share individual participant data that underlie the results reported in this article (after deidentification), including the study protocol and statistical analysis plan. Data availability will begin 9 months after publication and will be available 36 months after publication. To gain access, data requestors should submit a proposal to medicalinformation@karyopharm.com. Proposals will be reviewed by an independent review committee identified for this purpose.

## Abbreviations

ASCT: Autologous stem cell transplant
CR: Complete response
DLBCL: Diffuse large B cell lymphoma
HR: Hazard ratio
ORR: Overall response rate
OS: Overall survival
PR: Partial response
R-CHOP: Rituximab and cyclophosphamide, doxorubicin, vincristine, and prednisone
R-ICE: Rituximab and ifosfamide, carboplatin, etoposide
R-IPI: Revised International Prognostic Index
RR: Relapsed/refractory
XPO1: Exportin-1
