# Supplementary material for: Survival among patients with relapsed/refractory diffuse large B cell lymphoma treated with single-agent selinexor in the SADAL study
Source: J Hematol Oncol. 2021 Jul 16;14:111. doi: 10.1186/s13045-021-01122-1 (PMC8283921; doi:10.1186/s13045-021-01122-1)
Supplement: Supplementary file 1 — Additional file 1. Supplemental Material. [file 13045_2021_1122_MOESM1_ESM.docx]

**Survival Among Patients with Relapsed/Refractory Diffuse Large B-Cell Lymphoma Treated with Single Agent Selinexor in the SADAL Study**

Marie Maerevoet, Josee. M Zijlstra, George Follows, Rene-Olivier Casasnovas, J.S.P Vermaat, Nagesh Kalakonda, Andre Goy, Sylvain Choquet, Eric Van Den Neste, Brian Hill, Catherine Thieblemont, Federica Cavallo,

Fatima De la Cruz, John Kuruvilla, Nada Hamad, Ulrich Jaeger, Paolo Caimi,

Ronit Gurion^18^, Krzysztof Warzocha^19^, Sameer Bakhshi^20^, Juan-Manuel Sancho^21^, Michael Schuster^22^, Miklos Egyed, Fritz Offner, Theodoros P. Vassilakopoulos, Priyanka Samal, Matthew Ku, Xiwen Ma,

Kelly Corona, Kamal Chamoun, Jatin Shah, Sharon Shacham, Michael G. Kauffman, Miguel Canales

**Supplemental Material**

- **Methods**
- **Table S1. Progression-free survival**
- **Table S2. Overall survival by response and relapse status**
- **Figure S1. Overall survival according to baseline characteristics.**
- **Figure S2. Overall survival according to relapse/refractory status.**

**Methods**

**Study design**

The SADAL study design has been previously described [1]. Patients were treated with 60mg selinexor orally twice weekly. The study initially included two treatment arms (60 mg and 100 mg twice-weekly doses of selinexor) with patients randomized 1:1. However, following the pre-specified interim analysis of the results after 63 patients, the 100 mg dose was discontinued in the protocol (version 7.0) on March 29, 2017, based on a similar ORR but a higher rate of adverse events (AEs) in the 100 mg twice-weekly dose arm.

The study was approved and performed in accordance with the International Conference on Harmonization, the Guidelines for Good Clinical Practice, appropriate regulatory requirements, and with approval of institutional review boards at individual enrolling institutions. All patients provided written informed consent before study start.

**Outcomes**

The primary endpoint was ORR defined as the proportion of patients who achieved CR or PR according to the 2014 Lugano criteria [2]. Secondary efficacy endpoints were duration of response (DOR) and disease control rate (DCR). Exploratory endpoints included PFS, OS TTP, PK and PD endpoints and subgroup analysis of DOR, DCR, OS, PFS in addition to Quality of Life (QoL) assessments.

**Patients**

The study enrolled patients over 18 years of age with pathologically confirmed de novo or transformed (from low grade NHL) DLBCL who had received at least 2 to 5 previous lines of systemic regimens with documented evidence of disease progression (according to 2014 Lugano criteria), an Eastern Cooperative Oncology Group (ECOG) performance status of 2 or less, platelet counts more than 75,000/μL and neutrophils >1,000/µL. In order to allow adequate recovery from prior therapies, patients had to have at least 60 days elapsed from their most recent systemic anti-DLBCL therapy if they had a PR or CR; all other patients had to have at least 14 weeks elapsed since the end of their most recent anti-DLBCL therapy.

Patients were ineligible if they had DLBCL with mucosa-associated lymphoid tissue [MALT] lymphoma, composite lymphoma (Hodgkin’s lymphoma [HL]+ non-Hodgkin’s lymphoma [NHL]), or DLBCL transformed from diseases other than indolent NHL, PMBL, known central nervous system lymphoma or meningeal involvement, or patients with active graft-versus-host disease after allogeneic stem cell transplantation.

The subgroups for the current analysis were selected by baseline characteristics including age, region, and Revised International Prognostic Index (R-IPI) baseline prognosis. Patients were grouped by prior therapies they received including the number of therapies and type of therapy such as ASCT treatment, lenalidomide, chemotherapy. No patients received prior CAR-T therapy. Refractory disease was defined as failure to achieve CR or PR of their most recent therapy. Subgroup analysis was also performed on patients that relapsed within six months or after six months following their last use of rituximab.

**Assessments**

Patients underwent response assessment of lymphoma according to the revised 2014 Lugano criteria. DLBCL status was assessed by PET and CT (or PET and MRI) every 8 weeks. All responses were assessed by a central independent radiological review and confirmed by an independent Oncologist Review. Safety was monitored through the assessment of adverse events AEs, concomitant medications, laboratory parameters, physical examinations, vital signs, weight, ECOG performance status, electrocardiogram, and ophthalmic examinations. All AEs and serious AEs (SAEs), regardless of relationship to study drug were recorded at every visit.

**Statistical analysis**

For categorical variables, summary tabulations of the number and percentage of patients within each category of the parameter is presented. A 2-sided 95% exact confidence interval (CI) is presented for ORR and DCR. For continuous variables the number of patients, mean, median, standard deviation, minimum, and maximum values are performed. Time-to-event data was summarized using Kaplan-Meier (KM) methodology using 25th, 50th (median), and 75^th^ percentiles with associated 2-sided 95% Cis. Log-rank test and Cox proportional hazards model was used to compare survival distributions between subgroups.

**References**

1. Kalakonda N, Maerevoet M, Cavallo F, Follows G, Goy A, Vermaat JSP, et al. Selinexor in patients with relapsed or refractory diffuse large B-cell lymphoma (SADAL): a single-arm, multinational, multicentre, open-label, phase 2 trial. Lancet Haematol [Internet]. Elsevier Ltd; 2020 [cited 2020 Jul 9];7:e511–22. Available from: www.thelancet.com/haematology

2. Cheson BD, Fisher RI, Barrington SF, Cavalli F, Schwartz LH, Zucca E, et al. Recommendations for initial evaluation, staging, and response assessment of hodgkin and non-hodgkin lymphoma: The lugano classification. J. Clin. Oncol. American Society of Clinical Oncology; 2014. p. 3059–67.

**Table 1****. Progression-free survival**

| **Patients (n)** | **Median PFS, months**  **(95% CI)** |
| --- | --- |
| **Age** |  |
| <70 (n=74) | 3.6 (1.9, 4.6) |
| ≥70 (n=60) | 2.6 (1.9, 6.2) |
| **Region** |  |
| North America (n=20) | 2.3 (1.7, 24.8) |
| Western Europe and Australia (n=91) | 3.6 (1.9, 5.5) |
| Central and Eastern Europe and India (n=23) | 2.0 (1.9, NR) |
| **Baseline Prognosis** |  |
| Very Good (R-IPI=0) or Good (R-IPI=1,2) (n=69) | 3.8 (2.6, 6.9) |
| Poor (R-IPI=3,4,5) (n=58) | 1.9 (1.6, 3.6) |
| **Number of prior systemic treatment regimens** |  |
| 2 (n=79) | 3.7 (2.1, 6.2) |
| >2 (n=55) | 2.1 (1.9, 3.8) |
| **Prior regimens** |  |
| No prior ASCT (n=94) | 2.1 (1.9, 3.7) |
| Prior ASCT (n=40) | 4.6 (1.9, 10.3) |
| Prior lenalidomide (n=10) | 3.7 (1.9, NR) |
| No prior lenalidomide (n=124) | 2.3 (1.9, 4.0) |
| **Response to last prior systemic therapy** |  |
| CR or PR (n=92) | 3.6 (2.1, 5.9) |
| Without a CR or PR (n=37) | 2.2 (1.9, 11.5) |
| **Relapse** |  |
| < 1 Year of Diagnosis of DLBCL (n=52) | 1.9 (1.7, 4.0) |
| ≥1 Year of Diagnosis of DLBCL (n=71) | 3.7 (2.3, 9.0) |
| < 1 Year to the First Systemic Treatment Regimen (n=71) | 2.1 (1.9, 3.7) |
| ≥1 Year to the First Systemic Treatment Regimen for DLBCL (n=46) | 3.8 (2.3, 24.8) |
| < 1 Year to the Last ASCT Therapy (n=23) | 3.8 (1.9, NR) |
| ≥1 Year to the Last ASCT Therapy (n=13) | 6.3 (1.9, 24.8) |
| < 6 Months to the Last Use of Rituximab (n=80) | 2.3 (1.9, 4.0) |
| ≥6 Months to the Last Use of Rituximab for DLBCL (n=39) | 3.6 (1.9, 7.0) |
| Never had a CR on Prior Therapy and Achieved CR on Selinexor (n=4) | 11.5 (5.6, NR) |
| Never had a CR/PR on Prior Therapy and Achieved CR/PR on Selinexor (n=4) | 12.2 (3.7, NR) |

ASCT, autologous stem cell transplant; CI, confidence interval; CR, complete response; NR, not reached; PD, progressive disease; PR, partial response; SD, stable disease.

**Table S2. Overall survival by response and relapse status**

| **Parameter** | **OS, months**  **(95% CI)** | **HR (95% CI);** | ***p value*** |
| --- | --- | --- | --- |
| **Response to last prior systemic therapy** |  |  |  |
| CR or PR (n=92) | 11.1 (7.8, 15.5) | 0.7182 (0.4403, 1.1715) | 0.1806 |
| Without a CR or PR (n=37) | 7.0 (3.1, 28.0) |  |  |
| **Relapse** |  |  |  |
| <1 Year of Diagnosis of DLBCL (n=52) | 5.2 (4.1, 12.6) | 1.5407 (0.9709, 2.4449) | 0.0641 |
| ≥1 Year of Diagnosis of DLBCL (n=71) | 13.1 (9.0, 28.0) |  |  |
| <1 Year to the First Systemic Treatment Regimen (n=71) | 7.8 (5.0, 16.9) | 1.1778 (0.7254, 1.9125) | 0.5087 |
| ≥1 Year to the First Systemic Treatment Regimen (n=46) | 9.8 (7.6, NR) |  |  |
| <1 Year to the Last ASCT Therapy (n=23) | 13.7 (7.8, NR) | 0.8821 (0.3459, 2.2495) | 0.7927 |
| ≥1 Year to the Last ASCT Therapy (n=13) | 9.1 (2.0, NR) |  |  |
| <6 Months to the Last Use of Rituximab (n=80) | 6.6 (4.6, 13.7) | 1.298 (0.7896, 2.134) | 0.2972 |
| ≥6 Months to the Last Use of Rituximab (n=39) | 10.9 (7.0, NR) |  |  |
| Never had a CR/PR on Prior Therapy and Achieved CR/PR on Selinexor (n=4) | NR (12.6, NR) |  | 0.0401 |
| Never had a CR on Prior Therapy and Achieved CR on Selinexor (n=4) | NR (NR, NR) |  | 0.0109 |

**Figure S1**


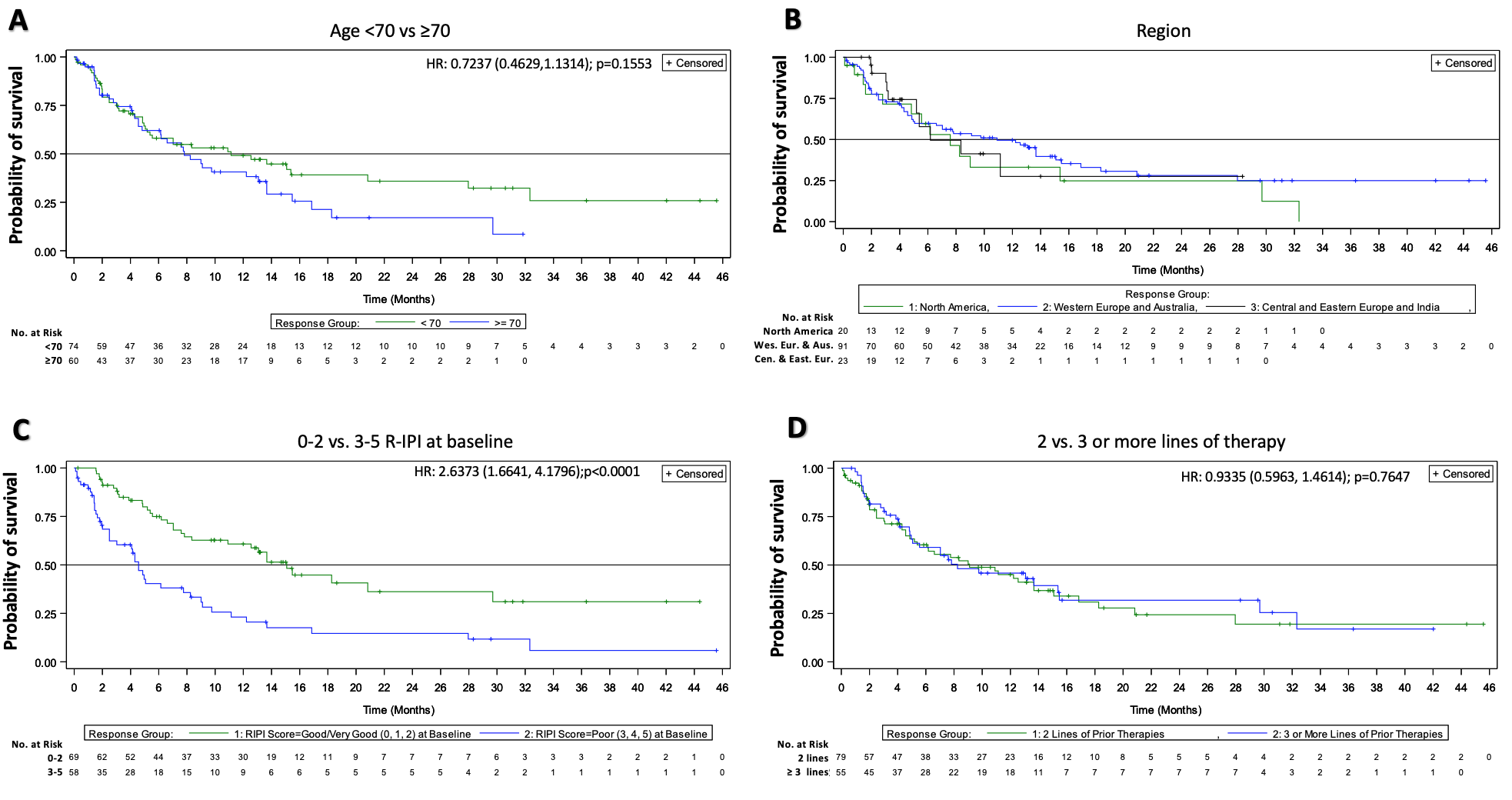


**Figure S1. Overall survival according to baseline characteristics.** Kaplan-Meier curves according to **A**. age; <70 and ≥ 70; **B**. region; North America, Western Europe and Australia, and Central and Eastern Europe and India; **C**. R-IPI Score; 0-2 and 3-5; and **D**. number of prior therapies: 2 lines and 3 or more lines.

**Figure S2.**


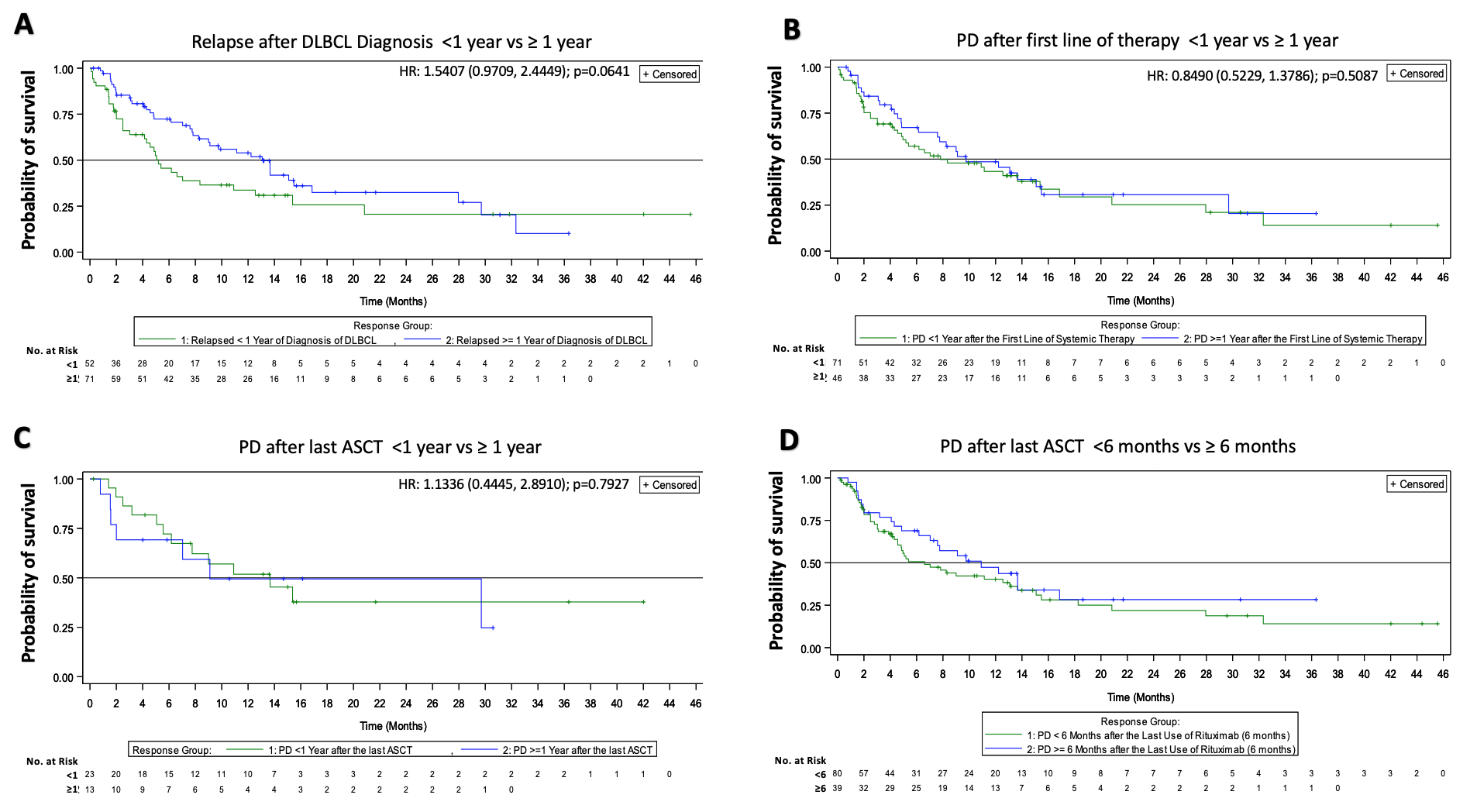


**Figure S2. Overall survival according to relapse/refractory status**. Kaplan-Meier curves for patients by **A**. time to relapse after DLBCL diagnosis; < 1 year (refractory) and ≥ 1 year; **B.** time to PD after first line of systemic therapy; < 1 year (refractory) and ≥ 1 year; **C.** time to PD after last ASCT treatment; <1 year (refractory) and ≥ 1 year. **D.** time to PD after last use of rituximab; < 6 months (refractory) and ≥ 6 months.
